# Spatial Association of Signaling Proteins and F-Actin Effects on Cluster Assembly Analyzed via Photoactivation Localization Microscopy in T Cells

**DOI:** 10.1371/journal.pone.0023586

**Published:** 2011-08-24

**Authors:** Chih-Jung Hsu, Tobias Baumgart

**Affiliations:** 1 Department of Chemistry, University of Pennsylvania, Philadelphia, Pennsylvania, United States of America; 2 Department of Chemical and Biomolecular Engineering, University of Pennsylvania, Philadelphia, Pennsylvania, United States of America; University of Pittsburgh, United States of America

## Abstract

Recognition of antigens by T cell receptors (TCRs) triggers cellular signaling cascades initiated by recruitment to the plasma membrane of numerous effector molecules to form signaling microclusters (MCs). Here we show that the method of high-resolution photoactivation localization microscopy (PALM) imaging can be used to analyze the spatial correlation between kinase ZAP70 and adaptor SLP76 MCs at the cell periphery and the effects of F-actin on MC assembly. We first determined the photophysical rate constants of Dronpa and tdEos fluorescence probes, which allowed us to optimize our dual-color PALM imaging method. We next analyzed the degrees of spatial association through determination of Mander's colocalization coefficients from PALM images, which revealed increasing spatial segregation of ZAP70 and SLP76 MCs at the cell periphery after initiation of signaling. We showed that this spatial segregation at the cell periphery occurred in parallel with the reduction of MC phosphorylation levels. Furthermore, we used Ripley's *K* function to analyze spatial randomness, and determined average radii of clusters as a function of activation time. The average radii of SLP76 and LAT MCs were found to decrease, whereas ZAP70 MC radii remained relatively constant. Finally, effects of F-actin depolymerization on MC morphology were studied by determining radial distributions of cluster circularity. Our data suggest that MC morphology is affected by actin polymerization. The quantitative analysis of sub-diffraction PALM images may provide a starting point for a molecular interpretation of cluster-cluster interactions and of the regulation of T cell signaling MCs by the cytoskeleton.

## Introduction

The engagement of the T cell antigen receptor (TCR) by antigen induces dynamic molecular rearrangement and leads to the translocation of kinases and adaptors to the plasma membrane of T cells [1], [2], [3]. Phosphorylation of TCR/CD3 complex is followed by the recruitment of zeta-chain associated protein kinase of 70 kDa (ZAP70) via its tandem Src-homology 2 domains. Once activated through the phosphorylation by lymphocyte-specific protein tyrosine kinase Lck, ZAP70 phosphorylates several adaptor proteins, including linker for activation of T cells (LAT) and SH2 domain containing leukocyte phosphoprotein of 76 kDa (SLP76) [4], [5]. Upon phosphorylation of LAT, SLP76 is recruited to the plasma membrane. These molecular events are essential elements of T cell activation, as suppression of membrane recruitment of downstream effectors by means of Src kinase inhibitors impairs T cell activation [1]. The phosphotyrosines of SLP76 in the N-terminal domain regulate binding to guanine-nucleotide-exchange factor VAV, the adaptor non-catalytic region of tyrosine kinase (NCK), and TEC-family kinase interleukin-2-inducible T-cell kinase (ITK) [6], [7], [8]. This molecular assembly results in the formation of signaling microclusters (MCs), which are continuously generated at the cell periphery [3], [9], [10]. It is currently agreed that these MCs are the sites for T cell activation [9], [10]. Tyrosine phosphorylation cascades and intracellular calcium flux are initiated soon after the formation of signaling MCs [1], [9], [10].

It has furthermore been shown that cytoskeletal components play a critical role in MC assembly and transport [10], [11], [12]. This MC transportation from the cell periphery was demonstrated to be essential to T cell signaling down-regulation [13], [14].

Fluorescence imaging techniques have been widely applied to monitor MC formation and transport upon T cell activation [1], [15]. T cells expressing signaling molecules fused with fluorescent proteins have been commonly placed on surface-immobilized anti-CD3 antibodies to trace dynamic assembly during early stages of activation. Quantitative characteristics of MCs have been investigated using fluorescence-based approaches including live-cell particle tracking [2], [15], fluorescence recovery after photobleaching (FRAP) [1], [15], and fluorescence resonance energy transfer (FRET) [8], [11]. Whereas these classical fluorescence imaging techniques yield information on the cellular distribution of signaling components, MC spatial clustering still lacks for quantitative analysis due to the diffraction limits in optical microscopy.

Here we adapted photoactivation localization microscopy (PALM) to visualize signaling MCs in Jurkat T cells activated by surface-immobilized ligands. The spatial association, cluster size and morphology, and interactions with peripheral F-actin were characterized in molecular detail. High resolution dual-color PALM images were obtained via use of a combination of two fluorescent protein variants: green-to-red photoswitchable tandem domain Eos (tdEos) and photoactivatable Dronpa [16], [17], [18]. This approach allowed us to visualize MCs with sub-optical resolution, to obtain spatial molecular coordinates, and to quantify cluster morphology.

The manuscript is organized as follows. We begin with an analysis of the photophysical reaction kinetics of our fluorescent probes, as is required for our dual-color PALM method. We then illustrate the advantage of PALM imaging in analyzing colocalization of signaling clusters. We proceed with an investigation of spatiotemporal details of MC formation, co-clustering, and degree of phosphorylation and, finally, study the effects of actin depolymerization on cluster morphology.

Our manuscript demonstrates that PALM imaging is a useful quantitative method to characterize the correlation between MC spatial association and MC phosphorylation and further to reveal the effects of cytoskeleton perturbation on MC features in T cells.

## Results and Discussion

### Photophysical rate constants of tdEos and Dronpa

**Figure 1:**
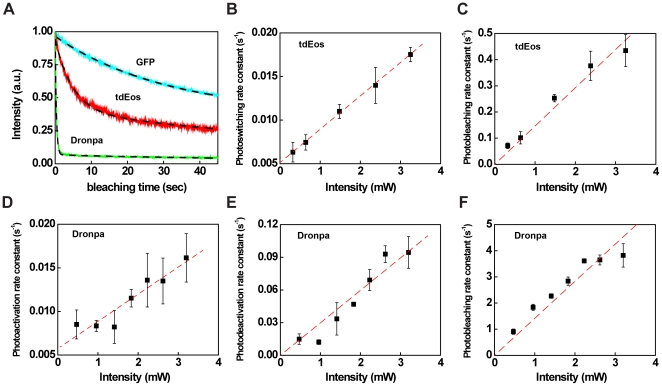
Photophysics of tdEos and Dronpa in cellular environments involve multiple rate constants. Photophysical rate constants of GFP, tdEos, and Dronpa were determined at the ensemble level in fixed cells by photobleaching in epi-illumination mode. (A) Normalized fluorescence trace of GFP (cyan), tdEos (red), or Dronpa (green) as a function of bleaching time was fitted to reaction schemes with varying numbers of photophysical rate constants (Eq. 1–9). The dashed line represents the best fit for one individual fluorescent protein type in one cell. (B) tdEos photoswitching rate constants and (C) photobleaching rate constants at different laser intensities. (D) Dronpa photoactivation rate constants, (E) reversible photodeactivation rate constants and (F) photobleaching rate constants at different laser intensities. Error bars represent the standard error of the mean. The number of analyzed cells was 5–7.

Dual-color high resolution PALM imaging has previously been performed via a combination of an irreversibly photoswitchable fluorophore and a reversibly photoactivatable fluorescent protein [17]. In order to be able to optimize dual-color PALM imaging for our purposes, we first determined photophysical properties of the fluorescent proteins tdEos and Dronpa in a cellular environment by fluorescence intensity measurements under continuous photobleaching during epi-fluorescence microscopy imaging (Fig. 1A) [19].

Cells transfected with tdEos constructs exhibited non-first-order photobleaching kinetics under 561 nm illumination, compared to photobleaching of GFP under 488 nm illumination, which followed first-order kinetics (Fig. S1A–S1B), as described before [19]. Therefore, we hypothesized that besides photobleaching, additional photophysical mechanisms contribute to the functional form of the experimentally observed fluorescence decay of tdEos (and Dronpa; see below) under continuous illumination [16]. We therefore evaluated if, in tdEos expressing cells, a reaction scheme including photoswitching and photobleaching (Eq. 1–3, see Materials and Methods) can improve fitting results compared to the consideration of photobleaching only. A fit of Eq. 3 to tdEos fluorescence traces led to significantly reduced residuals compared to first-order kinetics, which supported our assumption of photoswitching under 561 nm illumination (Fig. 1A–1B, and Fig. S1B). The non-zero intercept in tdEos photoswitching rate constant determination may indicate spontaneous photoactivation (Fig. 1B), as has been observed for Dronpa [16]. The photobleaching half time of tdEos ranging from 3.9 to 17.7 seconds in our measurement was comparable to reported values performed with similar illumination conditions for tdEos in the cellular environment at the ensemble level [20], [21] (Fig. 1C).

The PAGFP mutant Dronpa has been reported to exhibit reversible photodeactivation and spontaneous photoactivation [16]. We therefore adapted our photophysical reaction scheme to include photoactivation, photodeactivation, and photobleaching of Dronpa under 488 nm illumination (Eq. 4–9, see Materials and Methods). Like tdEos, a reaction scheme involving multiple photophysical rate constants yielded improved fits to Dronpa fluorescence decay (Fig. 1A, and Fig. S1C). The non-zero intercept in Fig. 1D confirmed the known spontaneous photoactivation of the Dronpa probe [16]. Compared to GFP and tdEos, Dronpa molecules exhibited a faster decay at the same laser intensity (Fig. 1A). Indeed, with 488 nm illumination, the photobleaching rate constant of Dronpa was significantly larger than photoactivation and photodeactivation rate constants at all illumination intensities (Fig. 1D–1F).

The investigation of probe photophysics just described has proven crucial for the cellular imaging studies discussed below. Due to the dominance of (irreversible) photobleaching over (reversible) photodeactivation at 488 nm illumination, we assume that most Dronpa molecules are imaged essentially only once during typical PALM imaging conditions. This allowed us to count fluorescent molecules contained in signaling microclusters. Furthermore, our observation of photoswitching of tdEos at 561 nm illumination allowed us to image tdEos without simultaneous 405 nm illumination. This is advantageous for two reasons: first, avoiding 405 nm illumination reduces photobleaching of Dronpa and tdEos, and second, we are able to stain a third component of interest (phosphotyrosine here, see below) with a blue fluorophore without interfering with green/red dual-color PALM imaging.

### Spatial distributions of signaling proteins in T cells

**Figure 2:**
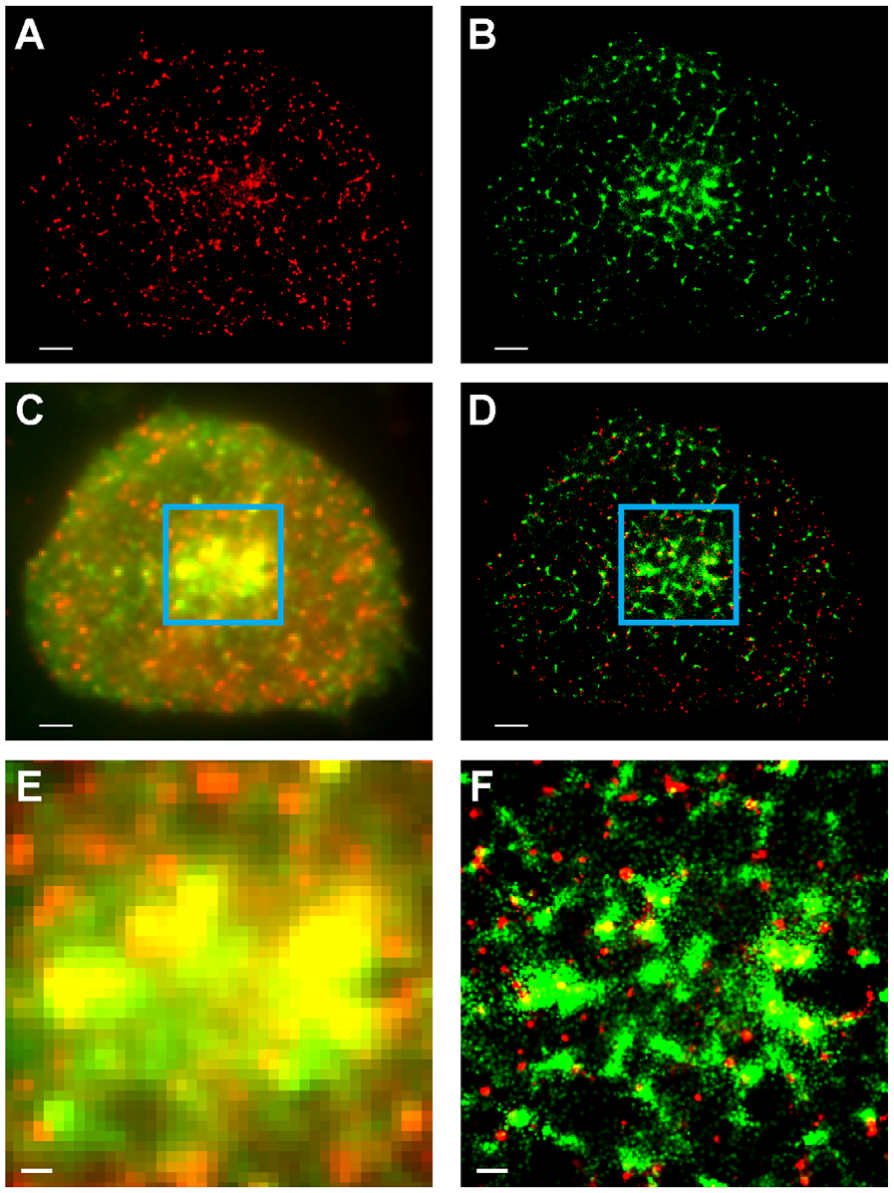
PALM allows sub-diffraction limit imaging of T cell signaling protein MC morphology. Membrane recruitment and MC assembly of protein tyrosine kinase ZAP70 and adaptor protein SLP76 occurs in response to TCR stimulation. (A) ZAP70tdEos PALM images obtained before Dronpa imaging. Coordinates were plotted as Gaussian spots as described in Materials and Methods. (B) SLP76Dronpa PALM images obtained after tdEos imaging. (C) Dual-color fluorescence wide-field TIRF and (D) PALM images. Scale bars in A–D: 2 µm. (E) Corresponding dual-color TIRF and (F) PALM images of an enlarged region boxed in C and D. High-resolution PALM images (D and F) display more discernible spatial features than wide-field TIRF images (C and E). Scale bars in E and F: 500 nm.

Human Jurkat T cells expressing ZAP70tdEos and SLP76Dronpa were stimulated via immobilized ligands and fixed for PALM imaging (see Materials and Methods). We selectively photoswitched tdEos by illuminating cells with 561 nm light only, while retaining Dronpa in a dark state. Figure 2A shows the PALM image of tdEos molecules localized and rendered as two-dimensional Gaussian spots (see Materials and Methods) [18], [22]. After the complete depletion of the pool of fluorescent tdEos molecules, Dronpa localization was performed, enabling us to obtain dual-color PALM images (Fig. 2A–2B, and 2D). Compared to TIRF, PALM images revealed significantly more detail allowing assessment of cluster spatial association and morphology (Fig. 2C–2D). Furthermore, in high protein density regions, only PALM imaging clearly showed that some apparently singular clusters in TIRF images were actually composed of multiple MCs with distinct morphologies (Fig. 2E–2F).

**Figure 3:**
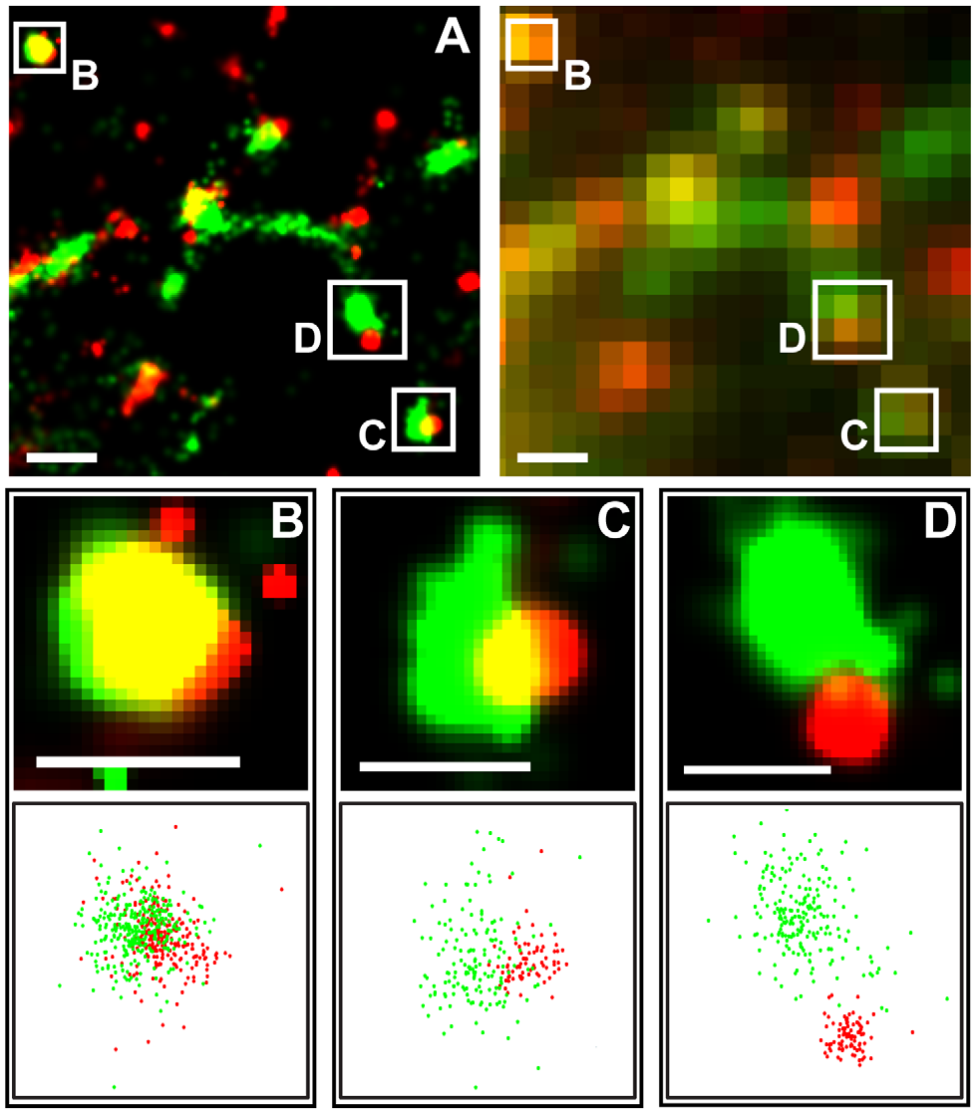
ZAP70 and SLP76 MC pairs visualized by PALM reveal distinct classes of spatial association. (A) Subcellular dual-color PALM images (left) resolve MCs invisible in wide-field TIRF (right) and reveal spatial association between ZAP70tdEos and SLP76Dronpa at the cell periphery (boxed regions). Scale bars: 500 nm. (B–D) Magnified views of distinct MC pairs of ZAP70tdEos and SLP76Dronpa boxed in (A) are shown as two dimensional Gaussian spots (top) or as molecular coordinates (bottom). The spatial categories defined in the main text are based on the Mander's colocalization coefficients M1 and M2 for tdEos and Dronpa, respectively. In case B, where the ZAP70tdEos MC is highly associated with SLP76Dronpa and M1 and M2 are 0.71 and 0.73, respectively; in case C, ZAP70tdEos MC is partially overlapped with SLP76Dronpa and M1 and M2 are 0.60 and 0.26, respectively; in case D, two MCs are only marginally associated and M1 and M2 are 0.21 and 0.05, respectively. Scale bars: 250 nm.

In response to TCR activation by immobile ligands, cytosolic protein tyrosine kinase ZAP70 and adaptor SLP76 were recruited to the plasma membrane where they formed signaling MCs (Fig. 2) [1]. Magnified PALM images revealed varying degrees of spatial overlap between peripheral ZAP70 and SLP76 MCs (Fig. 3A). Qualitatively, the degrees of association ranged from substantial colocalization to partial overlap, marginal association (Fig. 3B–3D), or full separation.

We used Mander's colocalization coefficients M1 and M2 [23], [24], [25] to quantify the degrees of molecular spatial association of MC pairs at the cell periphery. To depict the cellular distribution of MCs, the normalized cell radius 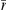 was defined here to range from zero at the cell center to one at the cell edge. The actin-enriched cell periphery region was operationally defined as the range of values for 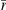 between 0.7 and 1.0. The colocalization coefficients reflect the amount of fluorescence of the colocalized area relative to the fluorescence from the total area covered by each clustered protein. Since ZAP70 and SLP76 MCs usually differ in total area, M1 and M2 are not expected to be equivalent. The spatial organization among ZAP70 and SLP76 was classified according to Eq. 10–11, which yield M1 or M2 equal to one for completely colocalized pairs and zero for isolated MCs. MC pairs were characterized as overlapping if they exhibited an M1 or an M2 value greater than 0.66 (Fig. 3B). The cluster pairs with both M1 and M2 less than 0.33 but larger than zero were defined as edge-connected (Fig. 3D). The rest of the MC pairs with non-zero colocalization coefficients were classified as partially overlapping (Fig. 3C).

**Figure 4:**
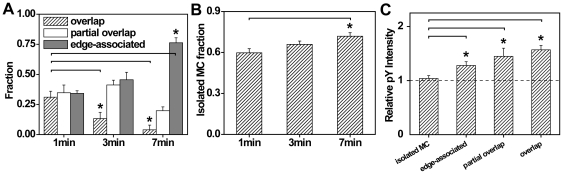
MC phosphorylation levels are related to the cluster spatial association. (A) The distribution of ZAP70 and SLP76 MC spatial association classes at the cell periphery shown at varying time after contact. The categories of MC pairs shown exhibit M1 and M2 larger than zero. (B) Increasing isolated (M1 = M2 = 0) MC fraction at the cell periphery as a function of time. (C) Relative phosphotyrosine (pY) levels quantified by the immunofluorescence of an anti-phosphotyrosine antibody at 3 min revealed an increasing phosphorylation extent in overlapping MC pairs. Error bars represent the standard error of the mean. The statistical significance (p≤0.05) is compared to the results at 1 min in A–B and to isolated MCs in C. The number of analyzed MC was greater than 300. The number of analyzed cells was 3–5.

To further investigate the distributions between these categories, we analyzed the proportion of each MC spatial association class at different contact times. At the early stage of stimulation (here cells were fixed after 1 minute of contact), MC association at the cell periphery was not predominated by any of the categories defined above (Fig. 4A). Between 1 min and 3 min, the average fractions of partial and edge-connected associations increased by 0.07 and 0.12, respectively, and the proportion of overlapping MCs diminished accordingly. At a later stage of stimulation (at 7 min after cell contact), the highly and partially overlapping fractions at the cell periphery diminished and edge-connected association exhibited an average fraction of 0.76, thus dominating over the other two categories. Meanwhile, the isolated MC fraction, of all analyzed ZAP70 and SLP76 MCs at the cell periphery, increased from 0.59 to 0.71 during the same period (Fig. 4B). The increasing proportions of edge-connected MC pairs and isolated MCs at the cell periphery were in accordance with the previously reported segregation of ZAP70 and SLP76 MCs at diffraction limited resolution [26].

From diffraction limited microscopy imaging, signaling MCs in T cells are known to be highly phosphorylated at the cell periphery, compared to the cell center [9], [14], [26]. To correlate the signaling functionality of MC pairs at the cell periphery with the degrees of spatial association identified by PALM imaging, we quantified MC phosphorylation levels among these categories. The relative phosphotyrosine (pY) extent here was defined as the ratio of the mean immunofluorescence intensity from MCs stained with 4G10 anti-phosphotyrosine antibody to the average intensity from an adjacent area but devoid of MCs. It has been observed that pY staining at 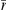 ranging from 0.7 to 1.0 shows negligible radial dependence, and that pY levels decrease from the cell periphery to the cell center (our data not shown, also see Refs. [9], [14], [26]). To investigate the correlation between pY and MC spatial association within the same cellular region, we compared the pY levels of ZAP70/SLP76 MC pairs at the cell periphery. Spatially associated ZAP70 and SLP76 MC pairs displayed significantly greater pY than isolated ones, which exhibited only marginally elevated intensity relative to the background (Fig. 4C and Fig. S2). Furthermore, our data suggest that MC phosphorylation levels were related to the degrees of MC spatial association (Fig. 4C). To our knowledge, this is the first report to demonstrate that MC phosphorylation occurs in parallel with the MC spatial association.

Preceding studies have shown that for T cells stimulated by surface-immobilized ligands, SLP76 MCs first nucleated around the stationary ZAP70 MCs and then moved to the center of the contact area [26]. This MC segregation from the cell periphery was proposed to be essential to T cell signaling down-regulation. The correlation between phosphorylation levels and MC spatial association in our experiment supports this model.

### MC clustering analysis

**Figure 5:**
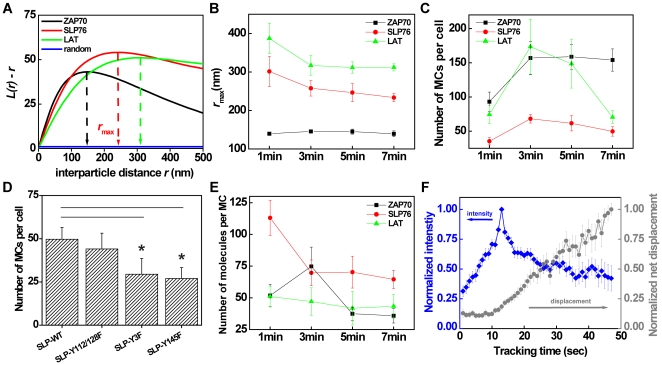
Compared to SLP76 and LAT, ZAP70 exhibits a unique clustering nature. (A) The linear transformation *L(r)-r* of Ripley's *K* function was computed from the molecular coordinates and plotted as a function of inter-particle distance *r* for representative cells at 3 min of contact, showing SLP76 (red), LAT (green), ZAP70 (black), or a simulated random distribution (blue). The amplitude of this function represents the degree of clustering. The distance corresponding to the maximum amplitude, *r*_max_, is depicted as dashed lines and is taken as a measure for the average MC radius. (B) The average *r*_max_ of SLP76 (red circles), LAT (green triangles) and ZAP70 (black squares) over different cells is plotted as a function of contact time. (C) Total number of MCs identified from PALM imaging is plotted as a function of contact time for SLP76 (red circles), LAT (green triangles) and ZAP70 (black squares). (D) Total number of MCs identified from PALM imaging for each SLP76 mutant is compared to SLP76-WT. (E) Total number of localized molecules per cluster is plotted as a function of contact time for SLP76 (red circles), LAT (green triangles) and ZAP70 (black squares). (F) Live-cell tracking of normalized SLP76GFP MC intensity (blue diamonds) and net displacement (gray circles) as a function of time. SLP76 single MC intensity and net displacement were normalized to the maxima of fluorescence intensity and net displacement, respectively. Data from individual MCs were synchronized at the peaks of SLP76 MC fluorescence intensity. Error bars represent the standard error of the mean. The statistical significance (p≤0.05) is compared to the result of SLP76-WT in D. The number of analyzed cells was 5–10.

Ripley's *K* function has previously been used in electron microscopy [27], [28], [29] and high-resolution fluorescence microscopy [30], [31], [32] imaging experiments to characterize spatial clustering and domain radius of nano-scale spatial organization. Ripley's *K* function provides a quantitative measure of the deviation of the observed distribution from a random distribution. Spatial clustering is usually reported in the form of a linear transformation *L(r) – r* of Ripley's *K* function (see Eq. 12–14, Materials and Methods). The positive amplitudes of *L(r) – r* found for all proteins examined here with spatial extension up to 500 nm indicated cluster formation (Fig. 5A). The distance corresponding to the maximum amplitude of *L(r) – r*, *r*_max_, was chosen to represent the average domain radius [27], [33], [34]. LAT MCs tended to display the largest *r*_max_, while ZAP70 exhibited the smallest value among the proteins investigated here (Fig. 5A–5B). The average *r*_max_ values of SLP76 and LAT MCs were reduced during the first five minutes after cell contact. The average *r*_max_ value of SLP76 decreased from 301 nm to 233 nm, and the average *r*_max_ of LAT was reduced from 387 nm to 312 nm. In comparison, the average *r*_max_ values for ZAP70 remained relatively constant between 140 nm to 145 nm (Fig. 5B). For control purposes, we analyzed clustering in cells adhering to non-stimulatory poly-L-lysine surfaces, where SLP76 displayed a shorter *r*_max_ and fewer MCs (Fig. S3).

We also analyzed the total number of MCs per cell as a function of time (Fig. 5C). The MC numbers per cell were similar but somewhat higher than the reported values [26], [35], [36], [37]. We attributed this discrepancy to the superior resolution of PALM imaging. From 1–3 min after contact, the total numbers of MCs increased for all proteins investigated here. This is likely a consequence of the cell contact area and increasing number of recognition sites presented to the stimulatory surface during this early stage [1], [13]. No significant changes in cell contact area after 3 min were observed in our fixed cell experiments or published live-cell images [38], suggesting steady membrane dynamics and constant number of recognition sites at the cell / surface contact. We note, however, that we cannot rule out an influence of cell spreading area on quantitative characteristics of MCs.

Furthermore, ZAP70 exhibited the longest temporal persistence among the proteins investigated here, in line with a confocal imaging study [1]. In contrast, the total number of LAT MCs peaked at 3 min after contact, after which clusters disappeared quickly, resulting in a relatively short temporal persistence (Fig. 5C).

The phosphotyrosines at the position of 112, 128, and 145 in the SLP76 N-terminal domain regulate binding to VAV, NCK, and ITK, respectively [39]. J14 (SLP76-deficient) cells reconstituted with a SLP76 mutant where three N-terminal tyrosines were replaced to phenylalanines exhibited impaired phospholipase Cγ1 (PLCγ1) phosphorylation, calcium flux and NFAT transcriptional functions [39], [40]. To further investigate the influence of these phosphotyrosines on SLP76 clustering, we constructed J14 cells stably expressing SLP76 mutants of Y145F, Y112/128F, or Y3F (Y112/128/145F) fused with the Dronpa probe. We observed that the Y112/128F mutant exhibited on average 44 MCs, and Y3F and Y145F mutants displayed an average number of 30 and 27 MCs which were significantly lower than the average number of 50 MCs in wild-type SLP76 cells. (Fig. 5D). This was consistent with earlier studies demonstrating that the tyrosine residue at the position of 145 was the most important to T cell activation [6], [39]. The reduction in the number of SLP76 MCs suggests that cooperative protein-protein interactions might stabilize MC structure and assembly during the stimulation [7], [8], [41]. This is also supported by the fact that ZAP70 could be backregulated by SLP76 in Jurkat T cells stimulated by immobile ligands [35]. ZAP70 MC formation and the fraction of cells with ZAP70 MCs are both significantly reduced in the absence of wild-type SLP76 and in the presence of a mutant of SLP76 (4KE) in Jurkat cells [35].

We further examined the total number of localized single molecules per cluster (Fig. 5E). For SLP76 MCs, this number quickly declined after 1 min of contact. These data were consistent with live-cell imaging experiments, where we found that SLP76 MCs displayed increasing fluorescence intensity, immediately after nucleation at the cell periphery. Upon reaching a fluorescence intensity maximum, SLP76 MCs translocated towards the cell center with decreasing fluorescence intensity (Fig. 5F). For LAT, a decrease in localized molecules per cluster as a function of time was also observed, although the reduction rate was less than for SLP76 (Fig. 5E). In comparison to SLP76 and LAT, ZAP70 exhibited unique changes in the number of localized particles per cluster over the same period in time. The number of molecules increased from 1–3 min after contact and dropped back to a lower level at later time (Fig. 5E). Overall, after 5 min, the numbers of localized particles for all proteins investigated here became steady and distributed between 50–80 for SLP76, 25–60 for LAT, and 25–45 for ZAP70. It has been reported that about 20 TCR molecules locate in one single MC [42]. Considering that multiple proteins bind to the TCR complexes, the numbers of localized molecules are similar to those determined for TCR.

Unlike LAT and SLP76, ZAP70, which directly binds to the immunoreceptor tyrosine-based activation motif (ITAM) of the TCR, exhibited an unexpected clustering nature including constant clustering radii and longer temporal persistence of MC structure. We speculate that these behaviors are related to the membrane receptor-ligand binding. We have observed that ZAP70 MC spatial and temporal dynamics are highly influenced by the engaged ligand mobility compared to SLP76, leading to more deformed MC morphologies on bilayer surfaces than on antibody-coated stimulatory cover glass (see below, Fig. S4). Furthermore, it has been shown that a fraction of SLP76 and LAT MCs activated by immobile ligands can be internalized from the cell membrane during stimulation [36], [43]. SLP76 and LAT MCs were found in endocytic vesicles but the exact mechanisms of internalization to date are unclear. Vale et al. have also recently observed phosphorylated TCR/CD3ζ complexes in the endosomal compartments [44]. These data indicate that protein dissolved or budded from the signaling complexes near the cell membrane may facilitate an uncharacterized signaling pathway involving the recycling machinery. The molecular distribution or clustering in this internalization process remains to be further investigated by the development of 3D high-resolution imaging [45], [46].

### F-actin modulation of MC assembly

**Figure 6:**
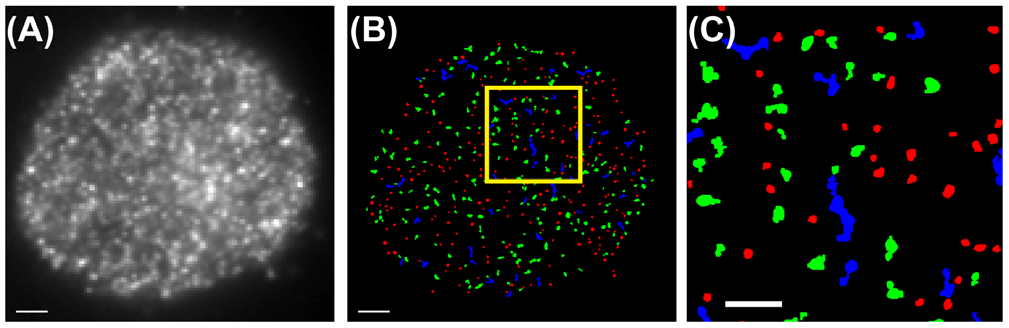
ZAP70 signaling MCs exhibit a variety of cluster morphologies unresolved in TIRF. (A) TIRF image obtained from Jurkat T cells expressing ZAP70tdEos activated by immobile ligands and fixed at 7 min after contact. (B) MC deformation is quantified by object circularity *C* in the corresponding PALM image. Individual MC morphology is depicted after area thresholding to eliminate unclustered features. MCs are classified into three groups according to the circularity with pseudocolor codes, red: *C*≥0.66, green: 0.66>*C*≥0.33, blue: 0.33>*C*. Scale bars in A–B: 2 µm. (C) A magnified view of the region boxed in B exhibits MC features from highly circular (red), intermediate (green) to irregular shapes (blue). Scale bar: 1 µm.

High-resolution fluorescence microscopy also allowed us to quantitatively describe MC shape, which is not resolvable through conventional TIRF imaging (Fig. 6A–6B). For automated cluster morphology characterization, PALM images were subjected to area thresholding to eliminate unclustered features (Fig. 6B). Circularity *C* was computed from the area and perimeter of the objects with areas above the threshold to represent the extent to which a MC resembles a perfect circle (*C* = 1) (Eq. 15). We classified MCs according to three groups defined as highly circular (*C*≥0.66), intermediate (0.66>*C*≥0.33) and irregular (0.33≥*C*) (color codes: red, green, and blue, respectively, in Fig. 6B–6C).

For ZAP70, we found MCs to exhibit a wide range of morphologies (Fig. 6B–6C). We note that it might be possible that small regular MCs fuse to form larger irregular ones. However, this fusion dynamic was not observed in live-cell TIRF imaging and remains to be characterized by live-cell PALM. At all the times investigated here, we have observed that SLP76 and LAT MCs exhibited less regular morphology than ZAP70 ones. The less regular features might potentially be influenced by the dynamic nature of SLP76 and LAT MCs compared to the less mobile ZAP70 MCs activated by surface-immobilized ligands. We tested this hypothesis by constructing a supported lipid bilayer surface with mobile stimulatory ligands. On the fluid membrane, ZAP70 MCs were observed to move centrally by live-cell tracking (our data not shown, also see Ref. [9]). Cells expressing ZAP70tdEos, SLP76Dronpa, or LATDronpa were placed on the bilayer surface and fixed at 7 min after stimulation to analyze MC morphology. In line with the hypothesized link between MC morphology and movement, ZAP70 MCs exhibited significantly more deformed morphologies on the fluid membrane than those stimulated by surface-immobilized ligands (Fig. S4A–S4C). However, SLP76 and LAT MCs displayed only moderate changes in circularity along the radial distribution (Fig. S4D–S4E).

**Figure 7:**
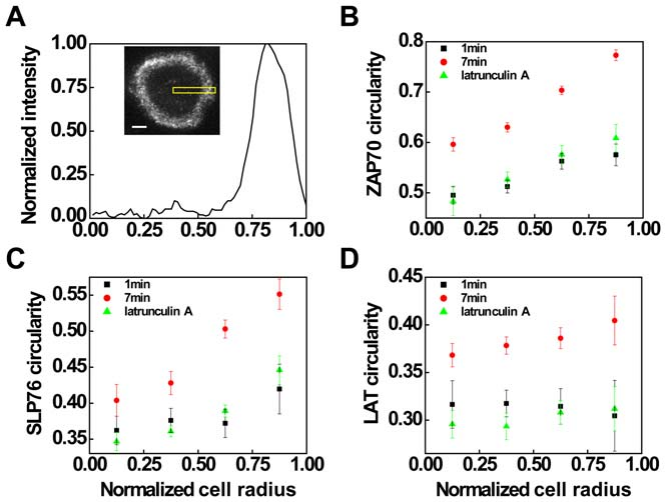
Signaling MCs are increasingly deformed along the inward radial direction. The sequential increase in MC circularity is actin-dependent. (A) Representative actinGFP radial distribution quantified by the epi-fluorescence profile highlighted in box (inset). The normalized cell radius 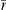 is defined as zero at the cell center and one at the cell edge. Scale bar: 5 µm. (B–D) The radial distribution of MC morphology is quantified by the mean circularity, using a binning size of 0.25 within a range of radial distances. Cells expressing (B) ZAP70tdEos, (C) SLP76Dronpa, or (D) LATDronpa were activated and fixed at 1 min (black squares) or 7 min (red circles) after contacting the stimulatory surfaces. Latrunculin A was added at 1 min after contact to inhibit F-actin polymerization and then cells were fixed at 7 min (green triangles). Error bars represent the standard error of the mean. The number of analyzed cells was 5–10.

F-actin has been shown to be essential for the assembly and movement of MCs [26], [47]. Depolymerization of F-actin by latrunculin A was shown to impair signaling and to inhibit the formation of new MCs at the cell periphery [47]. We thus hypothesized that MC morphology was influenced by F-actin polymerization. To test this hypothesis, we quantified the average MC circularity radial distribution and compared it to the actin density profile. Actin radial distributions were obtained as normalized fluorescence intensity, plotted as a function of radial distance (Fig. 7A). The actin radial distribution peaked at 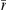 between 0.75–0.85 and was significantly reduced below the radial distance of 0.7 towards the cell center. At 7 min after contact, the inner ZAP70 MCs were more deformed (e.g. average circularity = 0.60 at 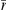 = 0.125, binning size = 0.25) than the outer ones (e.g. average circularity = 0.77 at 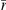 = 0.875) (Fig. 7B). For SLP76 and LAT MCs which displayed less regular morphologies, the radial circularity analysis revealed a similar decreasing tendency toward the cell center (Fig. 7C–7D). These data suggest that the spatial distribution of MC circularity conformed to the peripheral distribution of F-actin.

We next evaluated the influence of peripheral F-actin on the time-dependent MC circularity. From 1–7 min, the inner ZAP70 MCs increased by 0.1 in circularity, whereas the circularity of peripheral MCs significantly increased by 0.19 (Fig. 7B). For SLP76, during the same period, the peripheral *C* values increased from 0.42 to 0.55 compared to an increase by 0.04 of the inner ones (Fig. 7C). For LAT, over 7 min, the increase in *C* was 0.05 at 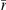 = 0.125 and 0.1 at 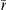 = 0.875, respectively (Fig. 7D). To investigate whether the larger circularity increases in peripheral MCs compared to the inner ones during the same period were affected by F-actin, cells were treated with 2 µM of latrunculin A at 1 min after contact to inhibit actin polymerization and then were fixed at 7 min to quantify resulting circularity changes. For all types of MCs studied here, depolymerization of F-actin severely deformed MCs compared to the untreated cells at 7 min after contact (Fig. 7B–7D). Cells treated with latrunculin A showed significantly more reduction of MC circularity in the periphery compared to the center, leading to a similar distribution with those fixed at 1 min and no latrunculin A at all 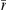 values. No significant enhancement in *C* was observed after the treatment with latrunculin A, suggesting that the sequential increase in *C* was actin-dependent. We verified this finding with SLP76 Y3F mutant where the interaction with VAV, NCL, and ITK were specifically altered, leading to disruption of actin polymerization. The cells expressing SLP76-Y3F were activated on surface-immobilized ligands and fixed at 7 min. The Y3F mutant exhibited a relatively flat radial distribution in circularity and the elevated MC circularity at the periphery was not observed (Fig. S5), possibly caused by the altered interaction between SLP76 and actin polymerization.

In live-cell imaging experiments, it has been shown that cell spreading involves dynamic rearrangement of cortical actin and the protrusion of lamellipodia at the cell periphery [38]. Cell expansion occurs over 1–2 minutes after contact [1], [13], [48]. This time period corresponds to the segregation of inner clusters from actin and cluster deformation at the smaller 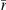 in our results. After 2–3 min of contact, the actin distribution reaches a steady state and only the peripheral MCs can be efficiently influenced [49], leading to the subsequent larger increases in circularity (Fig. 7B–7D).

To conclude, we have shown here that dual-color photoactivation and localization microscopy (PALM) can broaden the capacity to visualize nanostructure and quantify the MC spatial association in T cells. Our work provides a quantitative fluorescence-based approach to investigate the spatial distribution of signaling molecules and the interactions between signaling complexes and cytoskeletal components. Given the resolvable MC morphology under PALM imaging, future research studying the association of MCs with cytoskeletal features may help understand cluster transport mechanisms in T cells.

## Materials and Methods

### Plasmid constructs

Wild-type SLP76GFP cDNA and ΔMIGR vector [50] were gifts from G. Koretzky (Univ. of Pennsylvania). Wild-type ZAP70GFP cDNA was provided by L. Samelson (NIH). Dronpa N1 cDNA was purchased from MBL International Corporation (Woburn, MA). Tandem domain Eos (tdEos) N1 cDNA construct was from M. Davidson (Florida State Univ.).

The fluorescent protein chimeras containing Dronpa N1 and tdEos N1 we used in this work were obtained via standard cloning techniques. Prior to the restriction enzyme digestion, the endogenous nucleotide sequence ‘GGGCCTCGAGCCGC’ in ZAP70 was first mutated to ‘GGGCCTAGAGCCGC’ to eliminate the endogenous XhoI site (CTCGAG) using Site-Directed Mutagenesis (Stratagene, Santa Clara, CA). The cDNA segments encoding GFP in SLP76 and ZAP70 vectors were then digested with BamHI and NotI and ligated with Dronpa or tdEos inserts. These Dronpa or tdEos-containing plasmids were further digested with XhoI and NotI and ligated into the ΔMIGR vector.

### Cell lines, transfection, fixation, phosphotyrosine immunostaining, and cell culture

Wild-type human Jurkat T cells (JKT), SLP76-deficient Jurkat T cells (J14), J14 stably expressing SLP76GFP cells, LAT-deficient Jurkat T cells (Jcam2), and Jurkat T cells stably expressing actinGFP were gifts from G. Koretzky. Rat Basophilic Leukemia (RBL-2H3) cells used for photobleaching experiments were obtained from B. Baird (Cornell Univ.). All cell lines were maintained in a 5% CO_2_ humidified incubator (SANYO, Wood Dale, IL) at 37°C. Jurkat cells were cultured in phenol red-free RPMI 1640 medium (Invitrogen, Carlsbad, CA) with 10% fetal bovine serum (Thremo Scientific Hyclone, Logan, UT) and 20 mM glutamine without antibiotics. RBL cells were grown as monolayer culture in phenol red-free Minimum Essential Medium (Invitrogen, Carlsbad, CA) with 10% fetal bovine serum and 20 mM glutamine without antibiotics. Stably transfected cell lines were created using the ΔMIGR vectors mentioned above. Cells were harvested at the concentration of 1–2×10^5^/mL in culture medium for 24 hours and then resuspended at 20×10^6^/mL in serum-free RPMI medium prior to electroporation. One single electric pulse of 310 V and 20 ms was applied to a cuvette containing 500 µL of cell solution and 20 µg of cDNA using BTX electroporator (BTX Harvard Apparatus, St. Laurent, Quebec, Canada). After transfection and subsequent cell culture for two days, cells were sorted for Dronpa positive expression with a BD FACSVantage SE flow cytometer (BD Biosciences, San Jose, CA). After another week in culture, cells were sorted again to select stable transfectants. This process was repeated three times until cell populations with desired fluorescence levels were obtained.

For phosphotyrosine immunostaining, fixed-cell samples were permeabilized with 0.5% of Triton X-100 (Roche, Basel, Schweiz) and blocked with 10% BSA (Sigma, St. Louis, MO) in PBS [51]. Cells were incubated with diluted mouse anti-phosphotyrosine clone 4G10 primary antibody (Millipore, Billerica, MA) and then labeled with diluted Pacific Blue conjugated goat anti-mouse IgG secondary antibody (Invitrogen, Carlsbad, CA). For cell fixation and latrunculin A (Biomol International, Plymouth Meeting, PA) treatment, Jurkat T cells were first resuspended at 2–5×10^6^/mL in phenol red-free RPMI medium without serum. After one minute incubation in the stimulatory dish, cells were treated with 2 µM of latrunculin A and fixed with 4% formaldehyde (Fisher, Pittsburgh, PA) in PBS at varying times.

### Surface preparation

Immobilized stimulatory surfaces were made by applying 5 µL of 0.5 mg/mL anti-CD3 antibody (eBioscience, San Diego, CA) in 300 µL PBS (Thremo Scientific Hyclone, Logan, UT) to a clean Bioptechs delta T culture dish (Fisher, Pittsburgh, PA) with incubation at 4°C overnight. Supported lipid bilayer was formed by small unilamellar vesicle (SUV) fusion on the glass substrate. SUVs were composed of DMPC (1,2-dimyristoyl-*sn*-glycero-3-phosphocholine) (Avanti Polar Lipids, Alabaster, AL), additionally containing 0.2–2 molar percent of DSPE-PEG(2000)-biotin (1,2-distearoyl-*sn*-glycero-3-phosphoethanolamine-N-[biotinyl(polyethylene glycol)-2000]). The membrane was incubated for 30 min at room temperature with neutravidin (Thermo Scientific) for PALM imaging or neutravidin-Texas Red (Invitrogen) for FRAP. After the removal of unbound neutravidin, bilayer surface was added by biotinylated ligands and incubated at 4°C overnight. Membrane fluidity was characterized via FRAP experiments.

### Photobleaching measurements

J14SLP76GFP, J14SLP76Dronpa and RBL cells transiently transfected with SLP76tdEos were fixed with 4% formaldehyde in PBS. Cells were first photoactivated with 405 nm illumination and then continuously photobleached at 488 nm in the case of GFP and Dronpa or at 561 nm for tdEos. Fluorescence was collected via a cooled EMCCD camera (HAMAMATSU, Bridgewater, NJ) and recorded by HCImage software (HAMAMATSU, Bridgewater, NJ). Images were background corrected and then normalized to the fluorescence intensity maximum. Laser intensities were measured at the back aperture of the imaging objective.

### PALM Imaging

All Bioptechs delta T culture dishes and PBS used for PALM imaging were illuminated with a 100 W UV lamp (Ultra Violet Products, Upland, CA) for at least 30 minutes to reduce background fluorescence levels. Single molecules were imaged via a 60×1.45NA TIRF lens (Olympus, Center Valley, PA) on an inverted microscope system (IX71, Olympus, Center Valley, PA) using a 405 nm laser (5 mW, World Star Tech, Toronto, ON, Canada), 488 nm laser (50 mW, Coherent, Santa Clara, CA), and 561 nm laser (100 mW, CrystaLaser, Reno, NV) equipped with appropriate O.D. filters (Thorlabs, Newton, NJ). The system is equipped with one dichroic filter which reflects 405 nm, 488 nm and 561 nm (Chroma Technology, Bellows Falls,VT). In order to maximize spatial registration of the (sequentially collected) fluorescence images from tdEos and Dronpa, we avoided switching fluorescence cubes by using one dual-band emission filter which collects fluorescence at 500–545 nm and at 578–640 nm (Semrock, Rochester, NY). Images were acquired at a rate of 100 ms per frame. For single-color PALM experiments, Jurkat cells transiently transfected or stably expressing photoactivatable protein were used. Periodical short pulses of 405 nm illumination were controlled by a mechanical shutter and used for photoactivation. The 405 nm laser intensity was adjusted between 10–100 µW at the back aperture according to the protein expression levels. The 488 nm illumination laser was adjusted between 5–20 mW and continuously applied until depletion of photoactivatable molecules was achieved. For dual-color PALM, a stable Dronpa cell line was transiently transfected with a tdEos cDNA plasmid. The 561 nm laser was used to photoswitch and localize tdEos molecules. In order to reversibly switch Dronpa molecules to the dark state, the illumination intensity of the 488 nm laser was adjusted below 1 mW at the back aperture and continuously applied. Once the background fluorescence diminished, the 488 nm illumination was resumed to photoactivate and localize Dronpa molecules. As the localization density was reduced below the desired level (roughly 0.8molecules/µm^2^), the 405 nm laser was applied to increase the localization efficiency [16]. Obtaining one single PALM image usually took less than 10 min in our experiments. During this short acquisition time, the lateral drift of 5–8 nm characterized by using 50 nm fluorescent beads was comparable to previous high-resolution works [18], [52]. No stage drift correction was performed. The optical registration for dual-color PALM imaging was characterized via TetraSpeck microspheres (Invitrogen). The average deviation between red and green channels was 10–12 nm.

### Image analysis

We used MATLAB (Natick, MA) for image analysis. The background noise was first subtracted from raw fluorescence images. Background usually includes an evenly distributed component from the buffer or glass substrate as well as an uneven contribution from cellular autofluorescence [16]. The average pixel intensity of a frame was calculated to represent the even background. For the uneven background, a local morphological element was used to manipulate the imaging frame via image dilation and erosion. In our clustering features, the local morphological element was composed of a disk-shaped structure with a radius of 1 pixel. The frames were further subjected to three threshold values to achieve particle identification [16]. Any pixels above the first threshold T1 were selected and analyzed as a single object. Next, the object must have a minimum number of pixels above the second threshold T2 (T2 = 0.7×T1). This threshold was set to eliminate the occasional bright pixels from camera shot noise. Furthermore, the maximum number of pixels above the third threshold T3 (T3 = T1) was limited as well to reject objects which were too bright to represent single molecules. The particles that passed all three thresholds were then subjected to the following scheme to determine the position coordinates. The center of mass of each particle was used as initial values for the x-y coordinates of the molecule. A two dimensional Gaussian fit was performed, based on least-squares fitting to yield intensity amplitude and the localized coordinates with a localization uncertainty of 15–30 nm [22]. The localization algorithm also provided the number of localized particles within single MCs.

### Rendering of PALM images

PALM images used for display and for colocalization analysis consisted of pixels with dimensions equivalent to minimal localization uncertainty. Each localized molecule was plotted as a two dimensional Gaussian profile centered at the determined x-y coordinates and truncated at 5×5 PALM pixels to render a PALM image with zero background intensity [16], [18].

### Numerical image analysis

#### (1) Photophysical rate constants of GFP, tdEos and Dronpa

The photophysical rate constants were determined at the ensemble level in fixed cell samples by continuous photobleaching. GFP fluorescence intensity was fitted to single- exponential decay kinetics with one single photobleaching rate constant [19].

Equation 1 gives the entire photophysical reaction scheme of tdEos where *G* represents the fluorescent green state, *R* the fluorescent red state, and *B* the photobleaching state with photoswitching rate constant, *k_ps_*, and photobleaching rate constant, *k_b_*. The differential rate equation is given by Eq. 2. The rate equation can be integrated to yield Eq. 3. Fluorescence traces in response to a variety of laser intensities were fitted to Eq. 3 to obtain the rate constants using Matlab, as shown in Fig. 1A–1C. In the absence of the photoswitching term, the fitting quality was observed to decrease significantly (Fig. S1B).
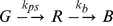
(1)

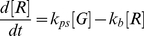
(2)

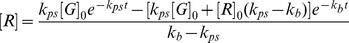
(3)


Equation 4 gives the entire photophysical reaction scheme of Dronpa where *I* represents the photoinactive state and *A* the photoactive state, respectively. Before photobleaching, the molecule Dronpa may first pass through a reversible process involving photoactivation with *k_pa_* and photodeactivation with *k_pda_*. The differential rate equation for Dronpa fluorescence is given in Eq. 5. The rate equation was integrated to yield Eq. 6 (with auxiliary Eq. 7–9). Fluorescence traces in response to a variety of laser intensities were fitted to equation 6 to obtain Dronpa rate constants, as shown in Fig. 1D–1F.
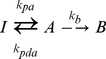
(4)

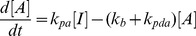
(5)

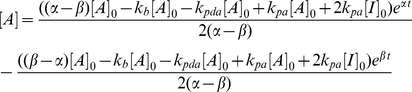
(6)

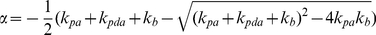
(7)

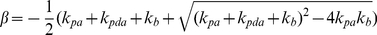
(8)

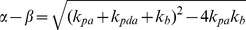
(9)


#### (2) Spatial overlaps between ZAP70 and SLP76

To quantify the MC spatial association, a dual-color PALM image with zero background intensity (Fig. 3B–D top panels) was used to compute Mander's colocalization coefficients M1 and M2:
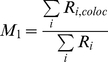
(10)

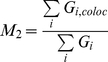
(11)where *R_i_* and *G_i_* represent the grey intensities of the red channel (tdEos) and green channel (Dronpa), respectively, and *R_i,coloc_* = *R_i_* if *G_i_*>0 or *R_i,coloc_* = 0 if *G_i_* = 0, and *G_i,coloc_* = *G_i_* if *R_i_*>0 or *G_i,coloc_* = 0 if *R_i_* = 0 [23], [24], [25]. In Eq. 10–11, summation occurs over pixel indices. MC pairs with M1 or M2 equal to zero were classified as isolated MCs. MC pairs with both M1 and M2 less than 0.33 (but larger than zero) were assigned to the edge-associated category. The highly overlapping category was defined as M1 or M2 greater than 0.66. The rest of the MC pairs belonged to the partially overlapping category. The cell periphery was defined as the actin-rich region in Fig. 7A, which here we took to range from 0.7 to 1.0 in normalized radial distribution.

#### (3) Relative pY intensity and live-cell J14 SLP76GFP tracking

Fluorescence intensity of phosphotyrosine antibodies was calculated after the subtraction of background intensity. The relative pY intensity was defined as the ratio of mean immunofluorescence intensity from MCs to an average value from an adjacent background area. Live-cell tracking in J14 cells stably expressing SLP76GFP was done as described previously [51].

#### (4) Ripley's *K*-function

Molecular coordinates were used to compute the spatial randomness of *n* particles in an imaging area *A*, within a certain radius *r* of any given molecule by Ripley's *K* function, *K*(*r*), as follows:
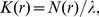
(12)where *N(r)* is the number of molecules within a distance *r* of a given particle, and *λ* is the mean particle density per unit area. An estimator of *K*(*r*) as suggested by Ripley was used [53]:
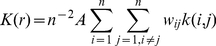
(13)where summation is over localized particles. If the distance between two particles *i* and *j* is less than *r*, then *k*(*i*,*j*) = 1, otherwise it is zero. The weighting factor for edge correction, *w_ij_*, was computed according to the method of Haase et al [54]. The linear transformation of *K*(*r*) was used to interpret the spatial randomness:
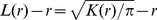
(14)The amplitude of the function *L(r) – r* will be negative for particles with anti-correlated distributions, zero ± the confidence interval (CI) for particles with a random distribution, and positive for clustering particles.

Significance tests of *L*(*r*) *– r* were performed by Monte Carlo simulation. The function of *L*(*r*) *– r* for a random distribution of *n* particles within the same image area was calculated for 300 time steps. The 99% confidence interval (CI_99_) was given by CI_99_ = 1.68·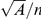 [53]. The CI_99_ values along with the simulated results were used to normalize *L(r) – r* function to yield ±1.

#### (5) Circularity (MC morphology)

High-resolution PALM images were first subjected to an area thresholding to remove non-clustered single molecules. The circularity, *C*, of each cluster was then computed as follows:
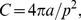
(15)where *a* is the area of one single MC, and *p* is the corresponding perimeter measured from a selected MC. The measurement of MC perimeter was based on an algorithm by K. Benkrid [55]. Briefly, the contour of an object is the sequence of links between the centers of adjacent edge pixels but not the count of the number of pixels. The individual contribution of each edge pixel to the perimeter depends on the path in which the contour passes through the pixel. Every single edge pixel is classified and weighted according to the path contribution and summed to represent the object perimeter. For example, a diagonal line across one single pixel contributes a distance increment equivalent to 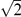 times the pixel dimension, while a straight line across one single pixel contributes a distance equivalent to the pixel dimension. Average values of *C* for one cell were first computed from a given number of MCs and multiple cells were analyzed to obtain the statistical results for the protein of interest.

#### (6) Radial distance determination

Cell center and radii were determined from TIRF images. The coordinates of individual MC were computed from PALM images. The radial distance of each single MC was computed as the distance from the MC mass center to the center of the cell. MC radial distance was further normalized to the cell radius to give 1.0 at the cell periphery and 0 at the center. For actinGFP radial distribution, actin density was characterized via the associated epi-fluorescence intensity profile which was normalized to its maximum.

## Supporting Information

Figure S1 Fluorescence traces of tdEos and Dronpa cannot be fitted with single exponential photobleaching decay. Cells transfected with GFP or Dronpa probes were continuously illuminated with 488 nm in Epi mode. Cells expressing tdEos probes were continuously illuminated with 561 nm laser. Normalized fluorescence trace of (A) GFP (cyan), (B) tdEos (red), or (C) Dronpa (green) as a function of bleaching time were fitted to single-exponential decays only involving photobleaching kinetics (black dashed line). Fitting residuals are shown in the bottom panels, comparing the fitting scheme only involving photobleaching (blue) to fits considering multiple rate constants (gray).
(TIF)

Figure S2 The relative extent of tyrosine phosphorylation is dependent on the spatial association levels of MC pairs at the cell periphery. (A) Subcellular dual-color PALM and (B) phosphotyrosine images of J14 Jurkat T cells stably expressing adaptor SLP76Dronpa (green), transiently transfected with kinase ZAP70tdEos (red) and stimulated by surface-immobilized anti-CD3 antibodies. Scale bars: 500 nm. The isolated ZAP70 or SLP76 MCs are highlighted in white boxes and spatially associated MC pairs are in purple boxes. The cell boundary is depicted in blue in B.
(TIF)

Figure S3 SLP76 exhibits a shorter *r*_max_ and fewer MCs in resting T cells. PALM images of J14 SLP76Dronpa cells placed on glass-coated (A) OKT3 or (B) poly-L-lysine (PLL) surfaces. Scale bars: 2 µm. (C) Analysis of Ripley's K function for a representative cell on OKT3 (black solid line) or PLL (red dashed line) surfaces.
(TIF)

Figure S4 ZAP70 MCs generated after stimulation by mobile ligands exhibit more deformed features compared to immobile ligands. (A) TIRF image of ZAP70tdEos cells stimulated by mobile biotinylated ligands anchored via neutravidin on a fluid bilayer containing polyethylene glycol biotinylated lipids. (B) MC deformation is quantified by object circularity *C* in the corresponding PALM image. Individual MC morphology is depicted after area thresholding to eliminate unclustered features. MCs are classified into three groups, red: *C*≥0.66, green: 0.66>*C*≥0.33, blue: 0.33>*C*. Scale bars: 2 µm. (C–E) The radial distribution of MC morphology is quantified by the mean circularity using a binning size of 0.25. The radial distance of each single MC is computed as the distance of the center of mass of the MC from the center of the cell. Cells expressing (C) ZAP70tdEos, (D) SLP76Dronpa, and (E) LATDronpa were activated by ligands immobilized on glass coverslips (red circles) or by mobile ligands on lipid bilayers (black squares). Cells were fixed at 7 min after contacting the stimulatory surfaces. Error bars represent the standard error of the mean. The number of analyzed cells was 5–10.
(TIF)

Figure S5Elevated MC circularity at the cell periphery is not observed in SLP76-Y3F mutant. The normalized cell radius 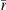 is defined as zero at the cell center and one at the cell edge. The radial distribution of MC morphology is quantified by the mean circularity, using a binning size of 0.25 within a range of radial distances. Cells expressing wild-type SLP76Dronpa (black squares) or Y3F SLP76Dronpa mutant (red circles) were fixed at 7 min and compared.(TIF)
