# Supplementary figures and images for: Spatial Association of Signaling Proteins and F-Actin Effects on Cluster Assembly Analyzed via Photoactivation Localization Microscopy in T Cells
Source: PLoS One. 2011 Aug 24;6(8):e23586. doi: 10.1371/journal.pone.0023586 (PMC3160965; doi:10.1371/journal.pone.0023586)

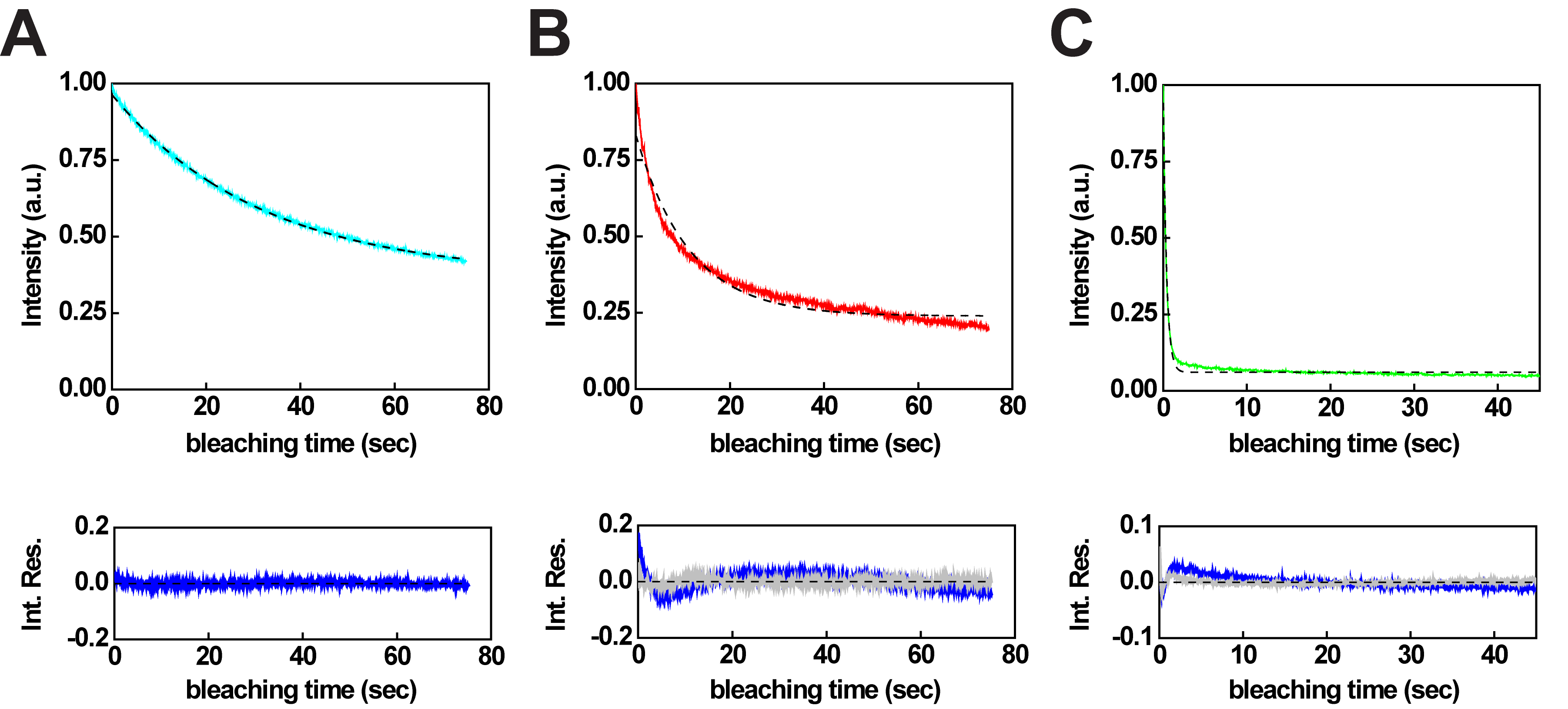

Supplement: Figure S1 — Fluorescence traces of tdEos and Dronpa cannot be fitted with single exponential photobleaching decay. Cells transfected with GFP or Dronpa probes were continuously illuminated with 488 nm in Epi mode. Cells expressing tdEos probes were continuously illuminated with 561 nm laser. Normalized fluorescence trace of (A) GFP (cyan), (B) tdEos (red), or (C) Dronpa (green) as a function of bleaching time were fitted to single-exponential decays only involving photobleaching kinetics (black dashed line). Fitting residuals are shown in the bottom panels, comparing the fitting scheme only involving photobleaching (blue) to fits considering multiple rate constants (gray). (TIF) [file pone.0023586.s001.tif]

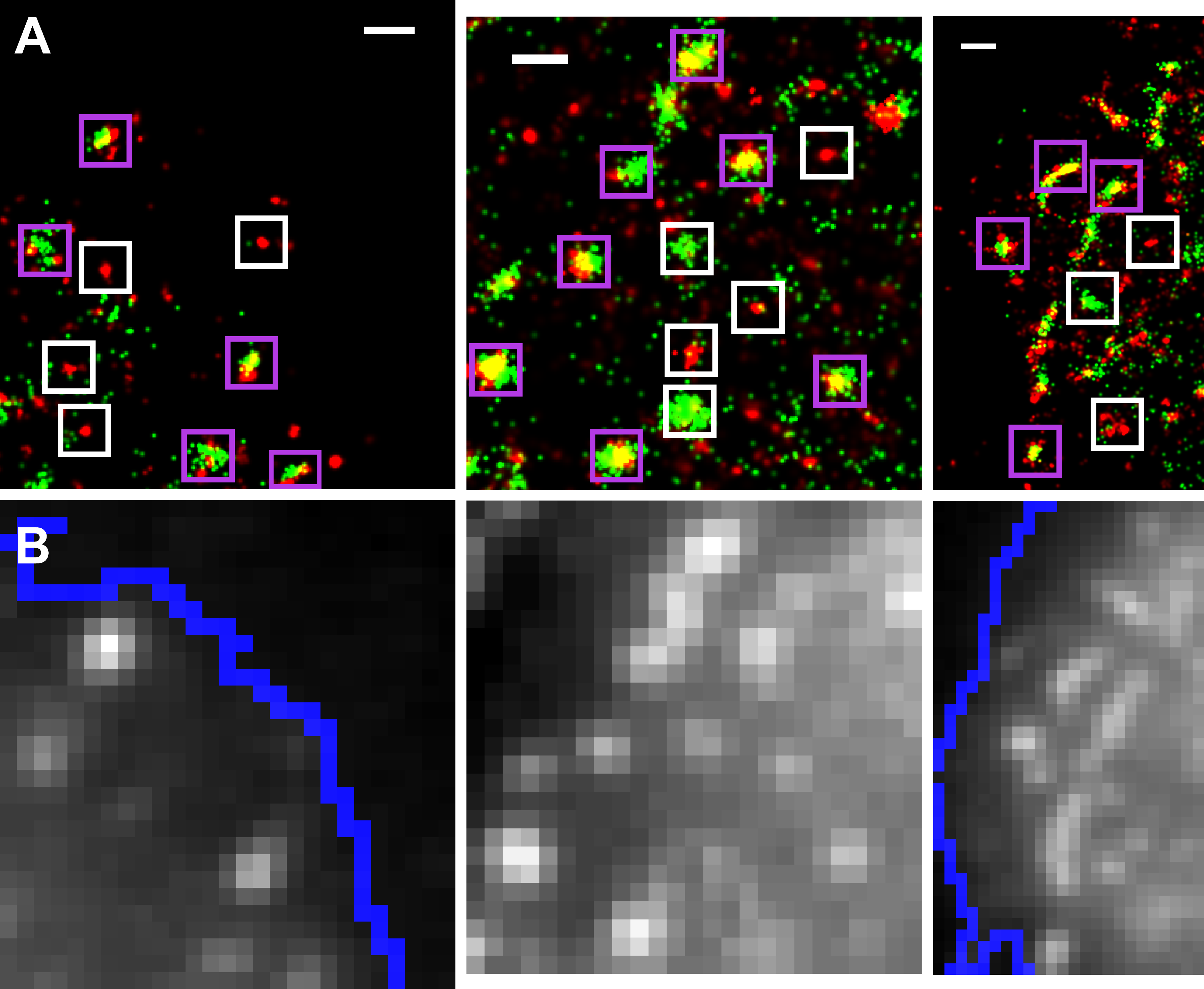

Supplement: Figure S2 — The relative extent of tyrosine phosphorylation is dependent on the spatial association levels of MC pairs at the cell periphery. (A) Subcellular dual-color PALM and (B) phosphotyrosine images of J14 Jurkat T cells stably expressing adaptor SLP76Dronpa (green), transiently transfected with kinase ZAP70tdEos (red) and stimulated by surface-immobilized anti-CD3 antibodies. Scale bars: 500 nm. The isolated ZAP70 or SLP76 MCs are highlighted in white boxes and spatially associated MC pairs are in purple boxes. The cell boundary is depicted in blue in B. (TIF) [file pone.0023586.s002.tif]

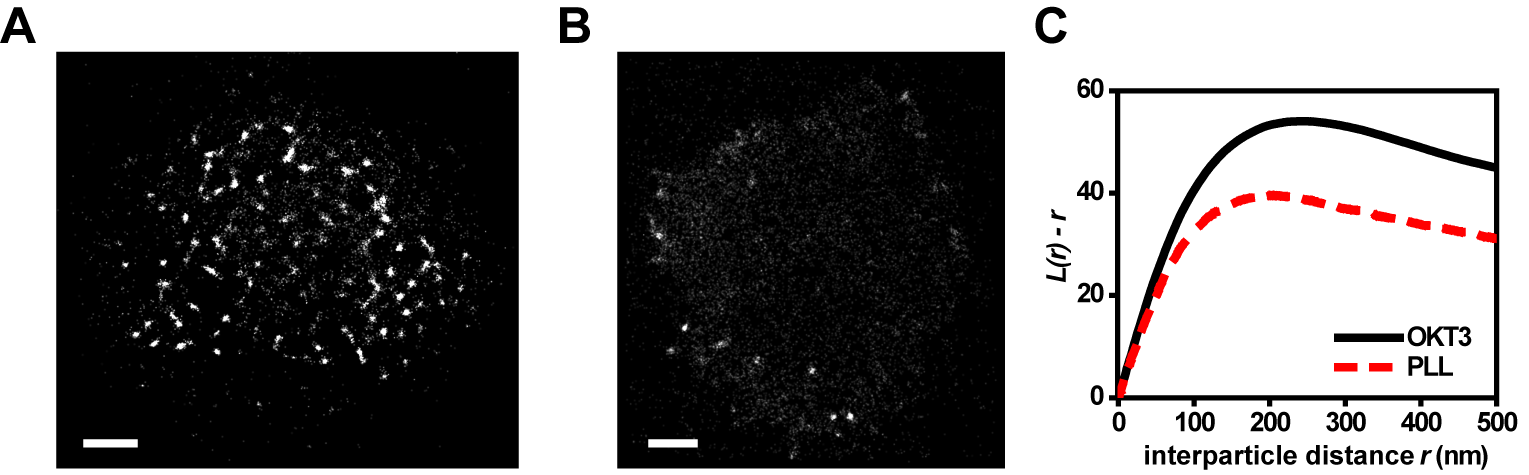

Supplement: Figure S3 — SLP76 exhibits a shorter r max and fewer MCs in resting T cells. PALM images of J14 SLP76Dronpa cells placed on glass-coated (A) OKT3 or (B) poly-L-lysine (PLL) surfaces. Scale bars: 2 µm. (C) Analysis of Ripley's K function for a representative cell on OKT3 (black solid line) or PLL (red dashed line) surfaces. (TIF) [file pone.0023586.s003.tif]

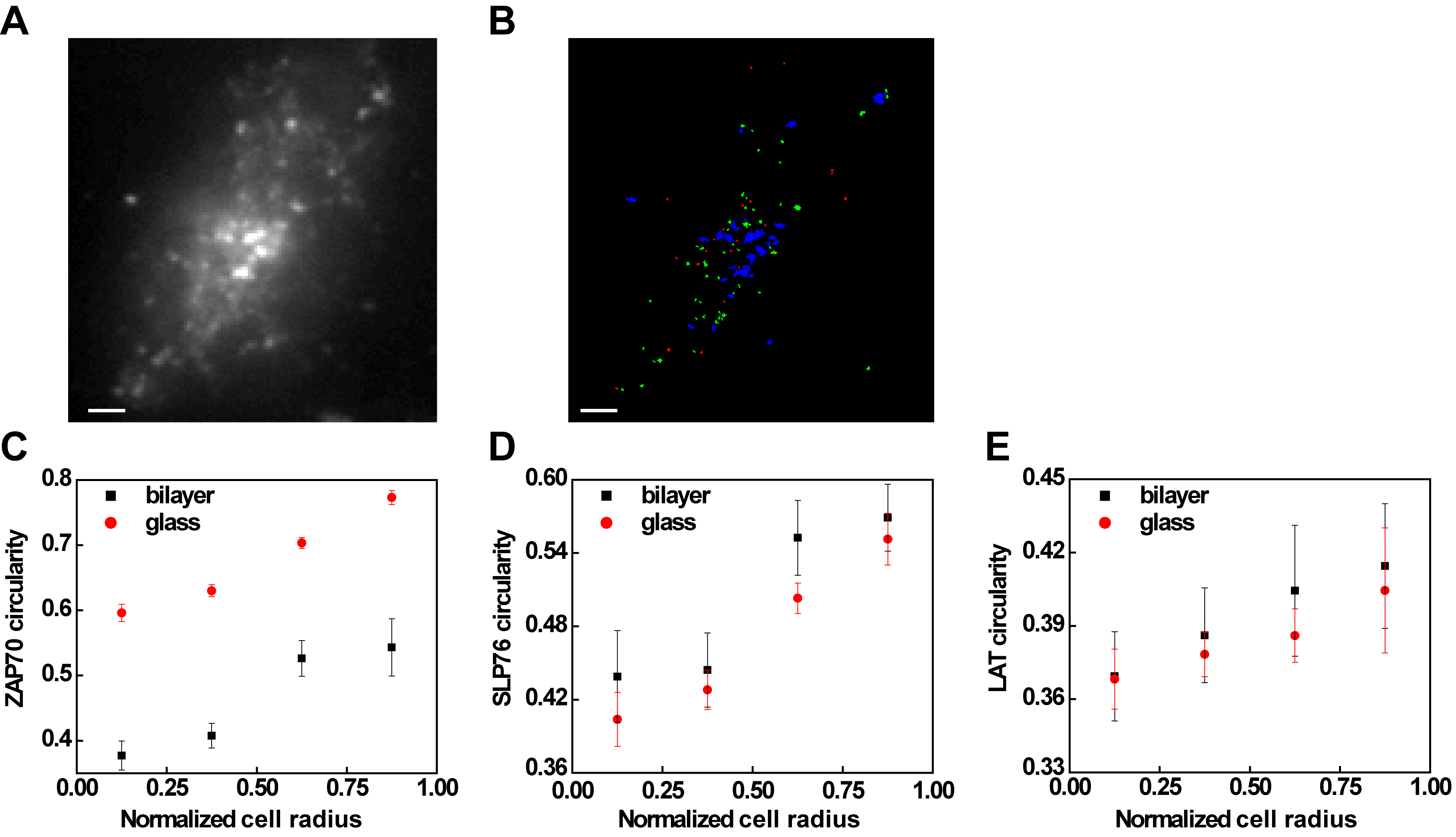

Supplement: Figure S4 — ZAP70 MCs generated after stimulation by mobile ligands exhibit more deformed features compared to immobile ligands. (A) TIRF image of ZAP70tdEos cells stimulated by mobile biotinylated ligands anchored via neutravidin on a fluid bilayer containing polyethylene glycol biotinylated lipids. (B) MC deformation is quantified by object circularity C in the corresponding PALM image. Individual MC morphology is depicted after area thresholding to eliminate unclustered features. MCs are classified into three groups, red: C≥0.66, green: 0.66>C≥0.33, blue: 0.33>C. Scale bars: 2 µm. (C–E) The radial distribution of MC morphology is quantified by the mean circularity using a binning size of 0.25. The radial distance of each single MC is computed as the distance of the center of mass of the MC from the center of the cell. Cells expressing (C) ZAP70tdEos, (D) SLP76Dronpa, and (E) LATDronpa were activated by ligands immobilized on glass coverslips (red circles) or by mobile ligands on lipid bilayers (black squares). Cells were fixed at 7 min after contacting the stimulatory surfaces. Error bars represent the standard error of the mean. The number of analyzed cells was 5–10. (TIF) [file pone.0023586.s004.tif]

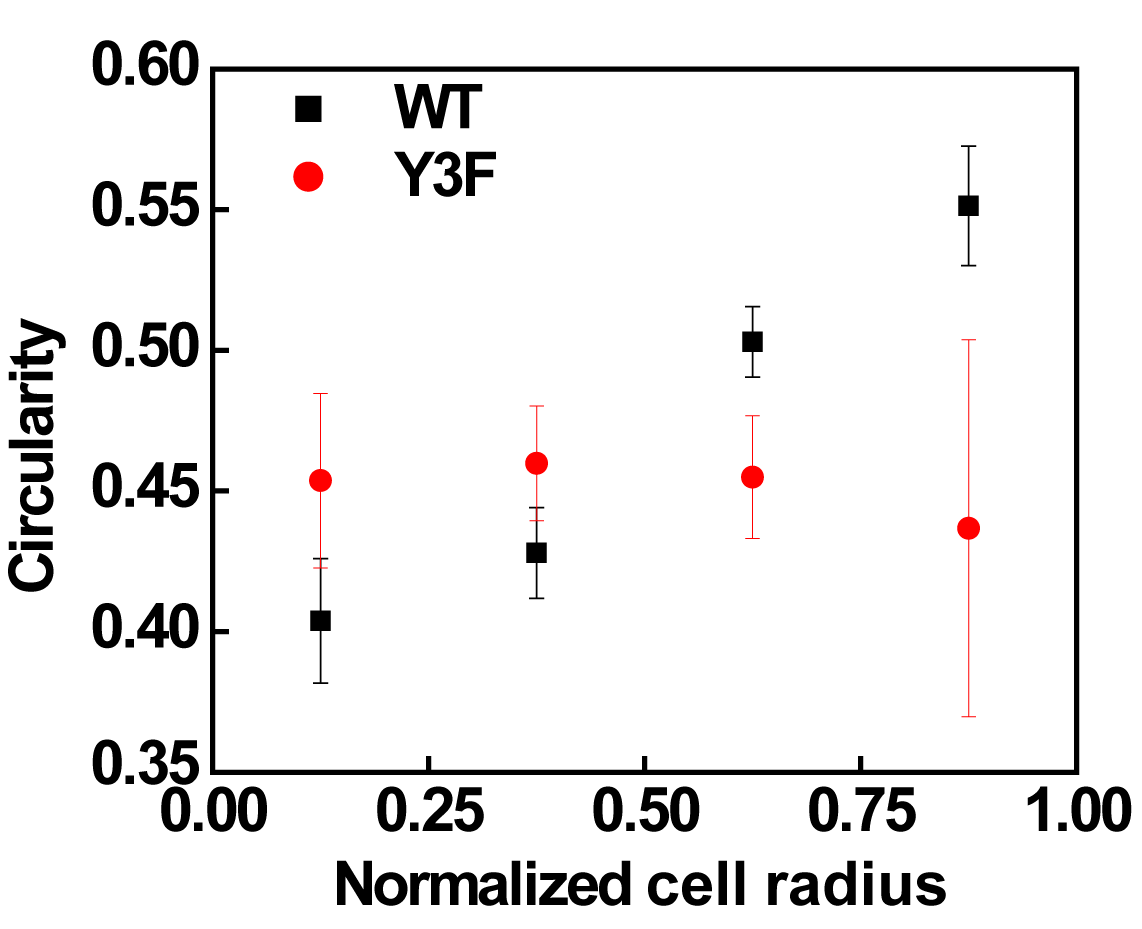

Supplement: Figure S5 — Elevated MC circularity at the cell periphery is not observed in SLP76-Y3F mutant. The normalized cell radius is defined as zero at the cell center and one at the cell edge. The radial distribution of MC morphology is quantified by the mean circularity, using a binning size of 0.25 within a range of radial distances. Cells expressing wild-type SLP76Dronpa (black squares) or Y3F SLP76Dronpa mutant (red circles) were fixed at 7 min and compared. (TIF) [file pone.0023586.s005.tif]
